# Updates in the perioperative and emergency management of non-vitamin K antagonist oral anticoagulants

**DOI:** 10.1186/s13054-015-0930-9

**Published:** 2015-04-29

**Authors:** David Faraoni, Jerrold H Levy, Pierre Albaladejo, Charles-Marc Samama, and the Groupe d’Intérêt en Hémostase Périopératoire

**Affiliations:** Department of Anesthesiology, Peri-operative and Pain Medicine, Boston Children’s Hospital, Harvard Medical School, Boston, MA 02115 USA; Department of Anesthesiology and Intensive Care, Duke University School of Medicine, Durham, NC 27710 USA; Department of Anesthesiology and Intensive Care Medicine, Grenoble University Hospital, Grenoble, 38043 France; Department of Anesthesiology and Intensive Care Medicine, Assistance Publique- Hôpitaux de Paris, Cochin University Hospital, Paris, 75181 France

## Abstract

Perioperative management of patients treated with the non-vitamin K antagonist oral anticoagulants is an ongoing challenge. Due to the lack of good clinical studies involving adequate monitoring and reversal therapies, management requires knowledge and understanding of pharmacokinetics, renal function, drug interactions, and evaluation of the surgical bleeding risk. Consideration of the benefit of reversal of anticoagulation is important and, for some low risk bleeding procedures, it may be in the patient’s interest to continue anticoagulation. In case of major intra-operative bleeding in patients likely to have therapeutic or supra-therapeutic levels of anticoagulation, specific reversal agents/antidotes would be of value but are currently lacking. As a consequence, a multimodal approach should be taken which includes the administration of 25 to 50 U/kg 4-factor prothrombin complex concentrates or 30 to 50 U/kg activated prothrombin complex concentrate (FEIBA®) in some life-threatening situations. Finally, further studies are needed to clarify the ideal therapeutic intervention.

## Non-vitamin K antagonist oral anticoagulants: new challenges

Direct thrombin inhibitors, dabigatran etexilate (Pradaxa®, Boehringer-Ingelheim Pharma GmbH, Ingelheim am Rhein, Germany), and direct factor Xa inhibitors, rivaroxaban (Xarelto®, Johnson and Johnson/Bayer HealthCare AG, Leverkusen, Germany) and apixaban (Eliquis®, Bristol Myers Squibb/Pfizer, Uxbridge, UK), are non-vitamin K antagonist oral anticoagulants (NOACs) increasingly used in the treatment of venous thromboembolism, prevention of cerebrovascular embolism in patients with atrial fibrillation, and thromboprophylaxis in patients undergoing orthopedic surgery [1]. Although the advantages of these new agents include rapid onset (2 to 4 hours) of action, in addition to a predictable anticoagulant effect without monitoring requirements, different clinical conditions can impair their pharmacokinetics and pharmacodynamics [2]. Despite published management perspectives, strategies are not yet clearly defined for perioperative management in patients treated with NOACs.

However, a consistent finding is that NOACs may have a lower bleeding risk. A recent report that included 27,419 patients treated for 6 to 36 months with dabigatran or warfarin reported that 1,034 patients had 1,121 major bleeding episodes during treatment or within 3 days of temporary or permanent discontinuation of anticoagulation [3]. The 30-day mortality after the first major bleed was 9.1% in the dabigatran group compared with 13.0% in the warfarin group, and dabigatran-treated patients required a shorter ICU stay compared with that in warfarin-treated patients. Using data from a prospective, non-interventional registry (The Dresden NOAC registry (NCT01588119), Dresden, Germany), including patients treated with oral anticoagulants in the region of Dresden in Germany, Beyer-Westendorf and colleagues [4] analyzed rates, management, and outcome of rivaroxaban-related bleeding. From 1,776 patients treated with rivaroxaban, 762 patients (42.9%) experienced 1,082 bleeding episodes within 3 days of discontinuation. Most episodes were classified as minor (58.9%), but 35.0% experienced clinically relevant bleeding, and 6.1% had major bleeding. The rates of major bleeding per 100 patient-years were 3.4 (95% confidence interval (CI) 2.6 to 4.4) for all patients, 3.1 (95% CI 2.2 to 4.3) for patients anticoagulated in the context of atrial fibrillation, and 4.1 (95% CI 2.5 to 6.4) for venous thromboembolism prevention. In case of major bleeding, surgical or interventional treatment was needed in 37.8% and prothrombin complex concentrates (PCCs) were administered in 9.1%. These results indicate that, in real life, rates of rivaroxaban-related major bleeding may be lower than with vitamin K antagonists (15 to 20%), and the outcome may, at least, not be worse.

In approximately 25% of patients receiving NOACs, treatment was interrupted at least once for surgery or another invasive procedure [5,6]. In addition, managing anticoagulation in the perioperative period is problematic because all anticoagulants can cause bleeding [7]. Despite their apparent safety compared with warfarin, perioperative management of patients treated with NOACs is now a routine challenge. In a recent international survey, we observed that physicians had limited knowledge about the perioperative management of patients treated with NOACs, and the management of emergency procedures [8]. The goal of this article is to briefly review current evidence, and propose an algorithm based on published information for the perioperative management of patients treated with NOACs.

## Preoperative management of patients treated with non-vitamin K antagonist oral anticoagulants

Preoperative management of patients treated with NOACs will be influenced by different factors that include: (i) the pharmacokinetic characteristics of the drug and the possible interaction with other treatments; (ii) patient comorbidities, especially renal function; and (iii) factors related to surgery considering both the timing (elective or urgent) and the bleeding risk of the procedure.

Dabigatran etexilate is a prodrug converted to an active component, dabigatran, after an esterase-mediated hydrolysis. This drug has a very low bio-availability (3 to 7%), and has a major renal mechanism for elimination (approximately 80%). Direct factor Xa inhibitors (rivaroxaban, apixaban) are primarily metabolized by the liver (65 to 70%), although renal excretion is also present. Clinicians should consider that the half-life of the three drugs is close to 12 hours in most patients [9]. Dabigatran elimination is most influenced by renal function, and preoperative interruption should be based on creatinine clearance (CrCl) calculated according to the Cockcroft and Gault formula [10]. Patients with moderate renal impairment (CrCl of 30 to 50 ml/minute) demonstrated >2-fold higher plasma levels compared with subjects with normal renal function (CrCl >80 ml/minute) [11]. Although the bleeding risk reported in the RE-LY trial was lower with dabigatran in the presence of normal renal function, the rates of major bleeding were higher as renal function deteriorated [12]. Renal function is less an issue with rivaroxaban and apixaban until severe renal insufficiency occurs [13]. Drug interactions are less important with NOACs than vitamin K antagonists, but the following interactions are relevant: aspirin, non-steroidal anti-inflammatory drugs, P-glycoprotein inhibitors (for example, amiodarone), CYP3A4 inhibitors (for example, phenotiazin), or inducers (for example, alcohol, carbamazepine) are at increased risk of abnormal metabolism. Notably, more than 40% of the targeted population, aged >75 years old, receive at least one of these drugs [14].

**Figure 1:**
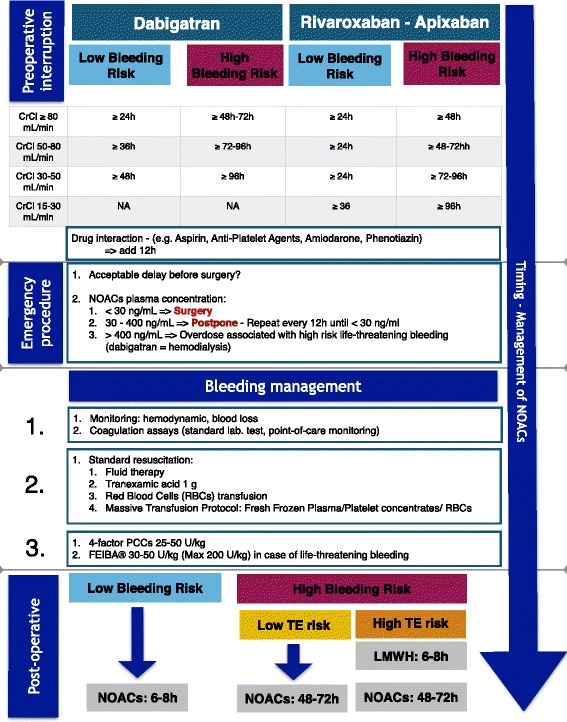
Proposed algorithm for peri-operative management of non-vitamin K antagonist oral anticoagulants. CrCl, creatinine clearance; LMWH, low molecular weight heparin; NA, not applicable; NOAC, non-vitamin K antagonist oral anticoagulant; PCC, prothrombin complex concentrate; TE, thromboembolism.

Different groups have published recommendations for perioperative management [15-19]. As recently recommended by the European Society of Cardiology [9], surgical procedures should be classified in three different categories: (i) interventions not requiring discontinuation of anticoagulation (for example, dental, ophthalmology, and superficial surgeries); (ii) intervention with low bleeding risk (for example, prostate or bladder biopsy, angiography, pacemaker implementation); or (iii) intervention with high bleeding risk (spinal or epidural anesthesia, lumbar diagnostic puncture, cardiothoracic surgery, abdominal surgery, major orthopedic surgery, liver biopsy, transurethral prostate resection, and kidney biopsy). Based on these guidelines [9,16,18], the three NOACs currently available should be stopped 24 hours before surgery with a low bleeding risk, and at least 48 hours and up to 96 hours before surgery with a high bleeding risk. In case of severe renal impairment (CrCl <30 ml/minute), interruption of direct factor Xa inhibitors should be delayed to 48 hours. For dabigatran, delays should be progressively prolonged based on CrCl (Figure 1) and potential laboratory testing should be considered to determine residual effects of the agents. It is important to mention that the use of dabigatran in patients with severe renal impairment (CrCl <30 ml/minute) is not recommended. Another way to manage these agents is to define a more conservative approach and to recommend the same interruption period for all the NOACs. With this approach, interruption should be 4 days in all patients and, in some cases (elderly, severe renal insufficiency), the interruption should approach 5 days. Additional studies are needed to optimize perioperative management.

In another analysis from the Dresden registry, Beyer-Westendorf and colleagues [6] assessed the management and the safety of perioperative NOAC use in 2,179 patients, of which 595 underwent 863 surgical procedures. Major cardiovascular events and major bleeding complications were low (1.0 and 1.2% after major procedures). Preoperative heparin bridging did not reduce the incidence of cardiovascular events, but was associated with an increased risk of major bleeding compared with the ‘no-bridging’ approach, especially in patients who underwent major procedures. This study reported that a short preoperative interruption was safe in patients who underwent either minor or major procedures. In case of major procedures, bridging therapy was not associated with a decreased incidence of cardiovascular event, while it significantly increased the bleeding risk. However, the benefit should always be balanced with the thromboembolic risk in high-risk patients. The role of additional coagulation testing, either specific (diluted thrombin time or specific anti-Xa activity) or non-specific (for example, activated partial thromboplastin time, and prothrombin time (PT)), in the preoperative management of NOACs for elective procedures needs to be determined.

Managing patients that require an emergency surgical procedure is an ongoing challenge. The timing between the last intake and the incident should be checked. Specific tests for NOACs effects are important to consider, and normal standard coagulation tests, PT or activated partial thromboplastin time, may not exclude the presence of clinically relevant concentrations of NOACs [20]. The effects of dabigatran can be assessed using specific diluted thrombin times (Haemoclot®) and direct factor Xa inhibitors require specific antifactor-Xa assays which are now available in the market. For both tests, results are expressed as ng/ml [21,22]. In recent published proposals, Pernod and colleagues and the GIHP group (Groupe d’Intérêt en Hémostase Périopératoire) [23] suggested that the measurement of the NOACs plasma concentration should be considered as the best way to assess the residual activity of the drug and to estimate the bleeding risk. If the plasma concentration is below 30 ng/ml, the surgery could be safely performed. Between 30 and 200 ng/ml, surgery should be delayed for at least 12 hours and testing repeated each 12 hours until the concentration reaches a safe level (<30 ng/ml). At concentration between 200 and 400 ng/ml, surgery should be delayed a minimal period of 24 hours. In patients with renal dysfunction treated with dabigatran, the relationship between concentration thresholds and time delays based on pharmacokinetics may be inaccurate. Dabigatran levels >400 ng/ml pose a major risk of uncontrollable hemorrhage, and hemodialysis should be considered. In the context of emergency procedures, the benefit associated with a surgery performed within a short delay should be balanced with the risk of major hemorrhage.

## Postoperative anticoagulation

Postoperative management of NOACs should be considered as restarting any anticoagulation agent in this setting. With appropriate hemostasis, NOACs potentially can be resumed 6 to 8 hours after minor procedures [9]. However, restarting full dose anticoagulation shortly after a procedure may increase the risk of postoperative bleeding. If this risk outweighs the risk of thromboembolic complication, full dose anticoagulation might be resumed 48 or 72 hours after the procedure [24]. In case of immobilization and increased risk of thromboembolic complication, thromboprophylaxis using low molecular weight heparins or unfractionated heparin should be started within 6 to 8 postoperative hours, whereas therapeutic anticoagulation with NOACs should be delayed for 48 to 72 hours after the procedure (Figure 1). Following major orthopedic surgery it may be appropriate to use prophylactic doses of NOACs until the patient can resume full dose anticoagulation.

## Intraoperative or postoperative bleeding management

There is limited information on the management of bleeding events in patients treated with NOACs. In a recent review of animal and human studies, Lee and colleagues [25] reported that although more than 15 trials assessed the effect of different treatment strategies to reverse the effect of NOACs, only two were performed in human volunteers, concluding that most of our experience came from animal bleeding models. In 2011, Eerenberg and colleagues [26] reported that administration of PCCs in healthy volunteers who received dabigatran 150 mg twice in one day improved laboratory parameters (PT and thrombin generation) but did not restore them to baseline. In another study performed in healthy volunteers, the authors reported that for both dabigatran and rivaroxaban, lower doses of activated PCCs (FEIBA® Baxter Healthcare, Westlake Village, CA,USA) appeared to be able to reverse the anticoagulant activity *ex vivo* in blood samples [27]. Although attempts were made to restore anticoagulant-induced bleeding using non-specific reversal agents, the current literature is suboptimal and clinical evaluations are needed regarding hemorrhagic situations. Only a few cases reported the efficacy of PCCs for urgent reversal of dabigatran in emergency situations [28].

Actually, preclinical data suggest that both prothrombin complex concentrates (three- or four-factor PCCs, 25 to 100 U/kg) and activated PCCs (FEIBA®, 20–80 U/kg), and recombinant activated factor VII (rFVIIa 90 μg/kg) can potentially correct the anticoagulation in case of massive bleeding after application of standard therapies (for example, fluid replacement, tranexamic acid) [29-33]. In a recent study performed in healthy volunteers, Levi and colleagues [34] confirmed that after receiving 4 days of rivaroxaban 20 mg twice in one day to obtain supra-therapeutic steady-state concentrations, both three- and four-factor PCCs partially reversed the anticoagulant effects of rivaroxaban in healthy adults. Administration of either the four-factor PCCs or the three-factor PCCs was safe and well tolerated, with no signs of thromboembolic events. Although discrepancy was reported between the effects of the two products on coagulation assays, the different effect observed for PT and thrombin generation are currently unclear.

Based on current literature, mainly from animal experimentations and preclinical studies, the off-label therapeutic use of both non-activated PCC (20 to 50 U/kg) and activated PCC (FEIBA®, 30 to 50 U/kg) may be considered for bleeding management in patients likely to have therapeutic or supra-therapeutic levels of NOACs (Figure 1) [35], although FEIBA®, and its high potential to overshoot thrombin generation, might be more effective in case of life-threatening bleeding; this benefit might be balanced with an increased risk of thrombosis [36]. Considering that no benefit was reported with the administration of rFVIIa alone, while this component could also be associated with a significant increase in the incidence of fatal thromboembolic complication [37], there is no evidence to support the use of rFVIIa.

Further specific antidotes are currently under development and will probably solve the issue of bleeding management [38,39]. Although hemodialysis could be an option in case of dabigatran-induced hemorrhage, little evidence exists and this solution should be limited to overdose with an increased risk of life-threatening hemorrhage [40]. In a recent study, Wang and colleagues reported that activated charcoal decreased the mean half-live of apixaban to 5 hours when charcoal was administered within 6 hours after the ingestion of the drug [41]. In the British Committee for Standards in Haematology guidelines, the administration of oral activated charcoal was recommended in bleeding patients who have taken a dose of dabigatran in the last 2 hours [19]. This short delay limits its use in our clinical practice.

## Conclusion

Perioperative management of patients treated with NOACs is an ongoing challenge. Specific recommendations have been made regarding management, but again are based on expert opinion (Figure 1). Due to the lack of good clinical studies involving adequate monitoring and reversal therapies, management requires knowledge and understanding of pharmacokinetics, renal function, drug interactions, and evaluation of the surgical bleeding risk. Consideration of the benefit of reversal of anticoagulation is important and for some low risk bleeding procedures it may be in the patient’s interest to continue anticoagulation. In case of major intra-operative bleeding where anticoagulation is thought to contribute substantially to the bleeding, standard therapy should include a multimodal approach including the administration of 25 to 50 U/kg four-factor PCCs or 30/50 U/kg activated PCCs (FEIBA®). Specific reversal agents/antidotes would be of value but are currently lacking. Finally, further studies are needed to clarify the ideal therapeutic intervention.

## Abbreviations

CI: Confidence interval
CrCl: Creatinine clearance
NOAC: Non-vitamin K antagonist oral anticoagulant
PCC: Prothrombin complex concentrate
PT: Prothrombin time
rFVIIa: recombinant activated factor VII
